# Structural basis of DNA recognition of the *Campylobacter jejuni* CosR regulator

**DOI:** 10.1128/mbio.03430-23

**Published:** 2024-02-07

**Authors:** Zhemin Zhang, Yuqi Yan, Jinji Pang, Lei Dai, Qijing Zhang, Edward W. Yu

**Affiliations:** 1 Department of Pharmacology, Case Western Reserve University School of Medicine, Cleveland, Ohio, USA; 2 Department of Veterinary Microbiology, College of Veterinary Medicine, Iowa State University, Ames, Iowa, USA; Dana-Farber Cancer Institute, Boston, Massachusetts, USA

**Keywords:** *Campylobacter jejuni*, CosR, oxidative stress, multidrug resistance, CosR-DNA complex, X-ray crystallography, cryo-electron microscopy

## Abstract

*Campylobacter jejuni* is a foodborne pathogen commonly found in the intestinal tracts of animals. This pathogen is a leading cause of gastroenteritis in humans. Besides its highly infectious nature, *C. jejuni* is increasingly resistant to a number of clinically administrated antibiotics. As a consequence, the Centers for Disease Control and Prevention has designated antibiotic-resistant *Campylobacter* as a serious antibiotic resistance threat in the United States. The *C. jejuni* CosR regulator is essential to the viability of this bacterium and is responsible for regulating the expression of a number of oxidative stress defense enzymes. Importantly, it also modulates the expression of the CmeABC multidrug efflux system, the most predominant and clinically important system in *C. jejuni* that mediates resistance to multiple antimicrobials. Here, we report structures of apo-CosR and CosR bound with a 21 bp DNA sequence located at the *cmeABC* promotor region using both single-particle cryo-electron microscopy and X-ray crystallography. These structures allow us to propose a novel mechanism for CosR regulation that involves a long-distance conformational coupling and rearrangement of the secondary structural elements of the regulator to bind target DNA.

**IMPORTANCE:**

*Campylobacter jejuni* has emerged as an antibiotic-resistant threat worldwide. CosR is an essential regulator for this bacterium and is important for *Campylobacter* adaptation to various stresses. Here, we describe the structural basis of CosR binding to target DNA as determined by cryo-electron microscopy and X-ray crystallography. Since CosR is a potential target for intervention, our studies may facilitate the development of novel therapeutics to combat *C. jejuni* infection.

## INTRODUCTION

*Campylobacter jejuni* is a leading foodborne pathogen that causes gastroenteritis in humans. This infection accounts for >400 million cases of diarrhea each year worldwide (1, 2). Enteritis is the prime clinical indicator of campylobacteriosis (3), which may include hematochezia, diarrhea, cramping, abdominal pain, fever, nausea, and vomiting. Extraintestinal infections such as bacteremia (4, 5), hepatitis (6), and pancreatitis (7) may also occur. In addition, some infected patients may develop postinfection complications, including reactive arthritis (8) and neurological disorders such as Guillain-Barré syndrome (2). As *C. jejuni* commonly resides in the intestinal tracts of most animals, eating undercooked poultry and meat or drinking raw milk are the usual ways to acquire this infection. Recently, the Centers for Disease Control and Prevention have classified antibiotic-resistant *Campylobacter* as a serious antibiotic resistance threat in the United States (9, 10) due to the rising prevalence of *Campylobacter* infections and their negative impact on public health.

In 1986, fluoroquinolones were approved as a key class of antimicrobials used for treating *Campylobacter* infections in humans (3). However, the high rate of fluoroquinolone-resistant *Campylobacter* infections has already limited the use of them as therapeutic drugs in patients (11, 12). Currently, macrolides such as azithromycin are the drugs of choice for antibiotic treatment of *Campylobacter* infections (3). Unfortunately, *C. jejuni* has become increasingly resistant to various clinically administrated antibiotics. Among the various mechanisms conferring antibiotic resistance in *Campylobacter*, the CmeABC efflux system plays a key role by reducing antibiotic accumulation within bacterial cells (13). Recently, a resistance-enhancing CmeB (RE-CmeB) variant was identified in *C. jejuni*, which was able to elevate the level of resistance to multiple antibiotics, including florfenicol, chloramphenicol, ciprofloxacin, erythromycin, and tetracycline (14). This RE-CmeB variant contains mutations within the CmeB multidrug efflux pump and, as a result, is functionally enhanced in antibiotic efflux and causes a sharp decrease in the susceptibility of the bacterium to various antimicrobials. Coupled with the T86I GryA amino acid substitution, the RE-CmeB variant can lead to a minimum inhibitory concentration (MIC) for ciprofloxacin ≥256 µg/mL (14), an MIC at least 64 times higher than the clinical breakpoint (15).

*C. jejuni* is often found to inhabit the intestinal tract of poultry, cattle, sheep, and swine. It is a microaerobic bacterium that grows in a low-oxygen environment found in the natural host. However, it also thrives in the human gut which is also a low oxygen environment, and under such conditions causes disease, whereas in the other animal hosts especially poultry, it is likely a commensal organism. Thus, it is sensitive to elevated oxygen concentrations (16) and utilizes several mechanisms in response to oxidative stresses (16, 17). Unlike other bacterial species, *Campylobacter* does not harbor SoxRS and OxyR regulatory systems for oxidative stress defense. Instead, *Campylobacter* utilizes other regulators such as PerR and CosR to modulate responses to oxidative stress (16). CosR is an essential gene and is vital to the survival of *C. jejuni*, which was illustrated by the inability to knockout the *cosR* gene in the organism (18). Two studies demonstrated the important role of CosR in the regulation of oxidative stress. It regulates the expression of a number of oxidative stress defense enzymes, including alkyl hydroperoxide reductase (AhpC), catalase (KatA), and superoxide dismutase (SodB) for the detoxification of reactive oxygen species (19, 20). In addition, there is strong evidence that *C. jejuni* CosR modulates the expression of the CmeABC multidrug efflux system (21, 22). Therefore, it has been proposed that the *C. jejuni* CosR regulator is an attractive target for the development of novel anti-*Campylobacter* drugs (23). Within the *cmeABC* promotor, there is a 21 bp DNA sequence TATTAACCAAAATTAAGATAT, containing the predicted regulator binding sites. This DNA sequence was found to be protected from DNase I cleavage by the CosR regulator (22), suggesting that CosR may specifically bind this DNA sequence for gene regulation.

CosR belongs to an OmpR/PhoB family of response regulators (24, 25). Its homologs are widespread in *ε-Proteobacteria*, such as *Campylobacter*, *Helicobacter*, and *Wolinella* (16, 26); however, CosR appears to be an orphan response regulator as a cognate sensor kinase was absent in *C. jejuni* (20). We previously determined the X-ray structures of the *C. jejuni* CmeB (27) and CmeC (28) membrane proteins, whose expression is regulated by the CosR (21, 22) and CmeR (29–31) regulators. We also used single-particle cryo-electron microscopy (cryo-EM) to resolve structures of the *C. jejuni* RE-CmeB multidrug efflux pump, both in the absence and presence of the antibiotics, ciprofloxacin, chloramphenicol, erythromycin, and hydrolyzed ampicillin (32). In addition, we used single-molecule fluorescence resonance energy transfer imaging to delineate the functional dynamics of this multidrug efflux pump (27). Based on these studies, we found that CmeB assembles as a trimer, and individual protomers of CmeB function independently to transport drugs within the trimer (27). Additionally, using X-ray crystallography, we determined the crystal structures of the CmeR regulator both in the absence and presence of bound bile acids (30, 31). In the present study, we aim to define the structural basis of CosR regulation in order to elucidate how CosR recognizes target DNA at the promotor region to control gene regulation. Here, we present the cryo-EM structure of apo-CosR and the X-ray structures CosR bound with target DNA. We determined that CosR assembles as a homodimer with each CosR protomer contributing to major conformational changes to facilitate DNA binding. These structures lead us to propose a mechanism that involves a long-distance conformational coupling between the N- and C-terminal domains and an induced fit event at the C-terminal domain to anchor the target DNA.

## RESULTS AND DISCUSSION

### Binding affinity of CosR to target DNA

**Fig 1:**
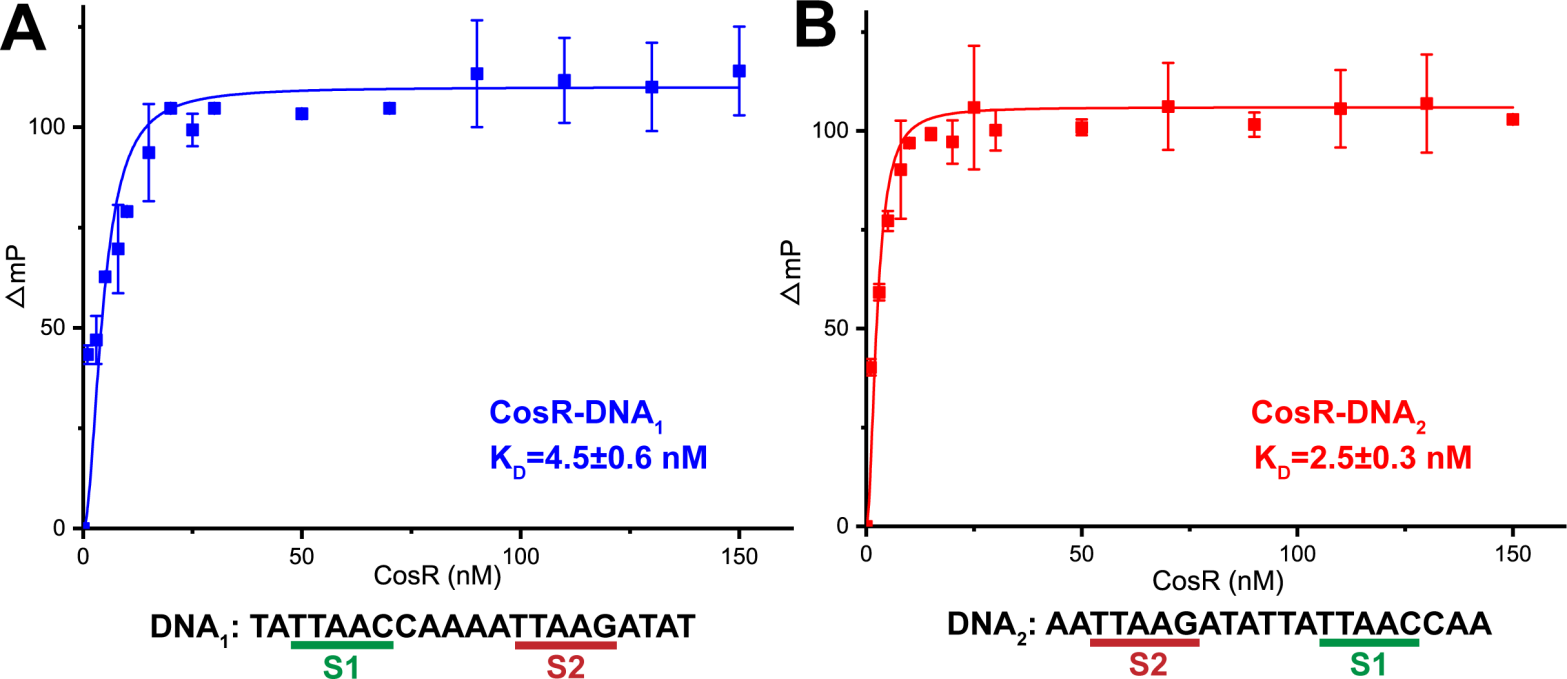
Fluorescence polarization of CosR with target DNAs. (**A**) The binding isotherm of CosR with the 21 bp DNA_1_ sequence containing the S1 and S2 sites. The K_D_ for this CosR-DNA_1_ binding was measured to be 4.5 ± 0.6 nM. (**B**) The binding isotherm of CosR with the 21 bp DNA_2_ sequence containing the S1 and S2 sites. The K_D_ for this CosR-DNA_2_ binding was measured to be 2.5 ± 0.3 nM.

Wild-type, full-length *C. jejuni* CosR with a 6xHis tag at the C-terminus was cloned into pET15b to generate the pET15bΩ*cosR* expression vector. This vector was used to overproduce and purify the CosR protein from *E. coli* BL21(DE3) cells. Previously, a gel electrophoresis mobility shift assay experiment suggested that CosR specifically binds to the *cmeABC* promotor region to regulate *cmeABC* expression (22). To confirm that CosR interacts with the *cmeABC* promotor, we used fluorescence polarization to quantify the interaction of the purified full-length CosR regulator with the 21 bp DNA sequence TATTAACCAAAATTAAGATAT (DNA_1_), which was previously found to be protected by CosR from DNase I cleavage in the *cmeABC* promotor region. Fig. 1A demonstrates the binding isotherm of CosR with 5 nM of the fluoresceinated 21 bp DNA duplex. The titration experiments indicate that CosR specifically binds this DNA sequence in the nanomolar range. The measured dissociation constant (K_D_) is 4.5 ± 0.6 nM. We also modified this 21 bp DNA by flipping the sequence AATTAAGATAT to the front of TATTAACCAA to form the new 21 bp DNA, AATTAAGATATTATTAACCAA (DNA_2_; see below). We found that CosR binds DNA_2_ tightly with K_D_ of 2.5 ± 0.3 nM (Fig. 1B).

### Cryo-EM structures of CosR

To elucidate the structural mechanism for CosR regulation, we first used the approach of single-particle cryo-EM. Purified full-length CosR (20 μM) was incubated for 2 hours with 10 µM of 40 bp double-stranded DNA (ds-DNA) sequence, which contains the 21 bp double-stranded DNA_1_, from the *cmeABC* promotor region. The increase in the size of the DNA chain may enhance the chance of obtaining the cryo-EM structural information. This CosR:DNA solution should contain a mixture-free CosR, free DNA, and the CosR-DNA complex. We recently developed a “build and retrieve” (BaR) cryo-EM methodology (33) that allows for the simultaneous identification and solving of structures of various biomacromolecules from a single impure, heterogeneous sample. BaR is an iterative methodology capable of performing *in silico* purification and sorting of images of several different classes of biomacromolecules within a large heterogeneous data set. We rationalized that BaR may allow us to observe images of both the DNA unbound and bound forms of CosR from a single cryo-EM grid. We, therefore, loaded the CosR:DNA sample onto a cryo-EM grid and collected single-particle cryo-EM images, even though the size of CosR (25 kDa per monomer) is quite small for the technique of cryo-EM.

**Fig 2:**
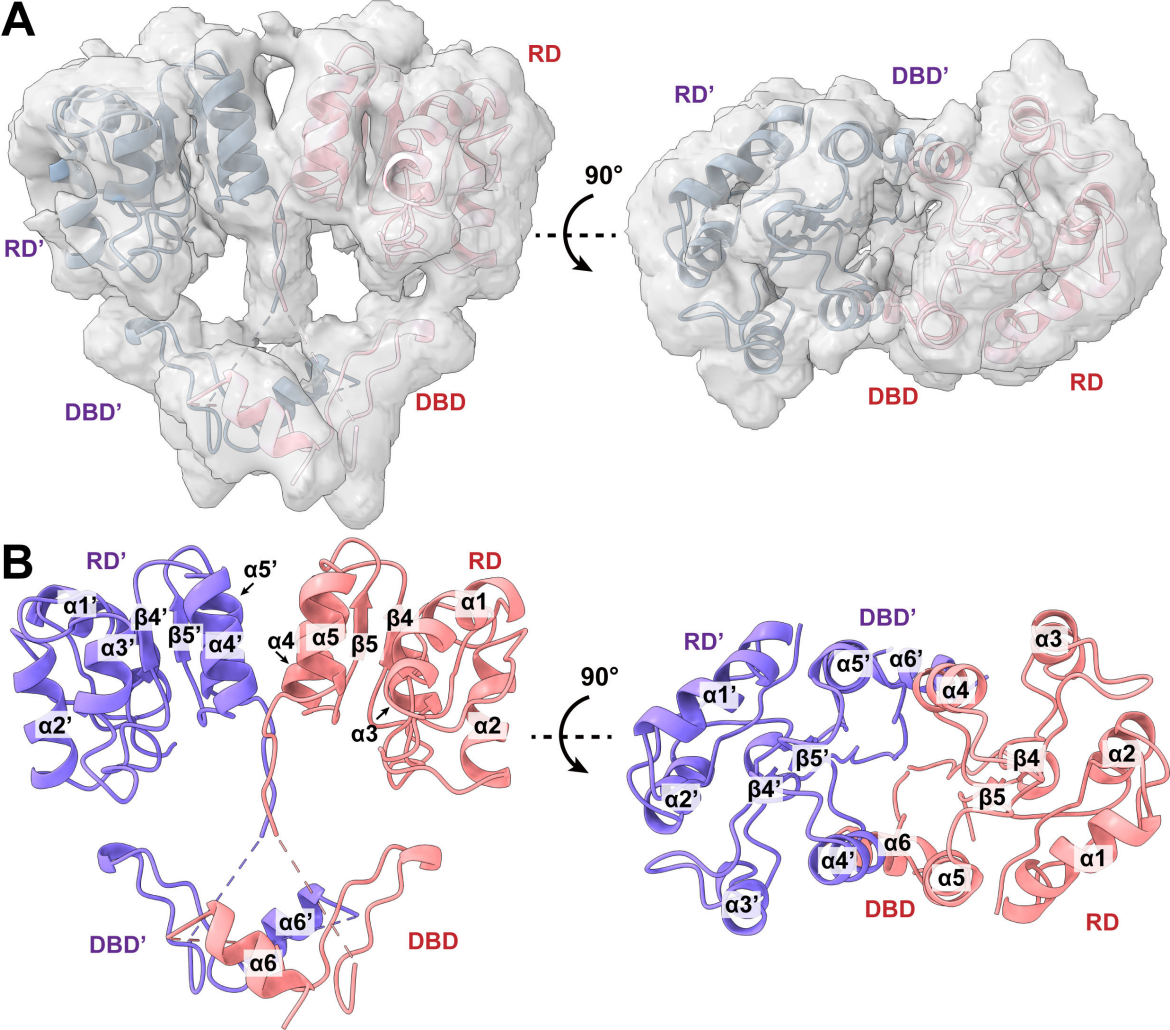
Cryo-EM structure of apo-CosR. (**A**) Cryo-EM density map of the CosR dimer. The data allow us to solve the cryo-EM of the apo-CosR dimer at a nominal resolution of 3.77 Å. (**B**) Ribbon diagram of the cryo-EM structure of apo-CosR. The two CosR molecules are colored pink and slate.

Extensive classification of the single-particle images indicated that there were three distinct classes of images coexisting in this sample (Fig. S1). Several iterative rounds of classifications allowed us to sort the images based on these classes. Interestingly, we indeed observed single-particle images of free CosR (apo-CosR), CosR-DNA, and free DNA. Three-dimensional reconstitutions of these particles allowed us to obtain cryo-EM maps of apo-CosR, CosR-DNA, and free DNA at nominal resolutions of 3.77, 7.71, and 6.61 Å, respectively (Fig. S1). The map of apo-CosR also enabled us to build a cryo-EM structural model of the apo-CosR regulator (Fig. 2A and B).

The apo-CosR regulator assembles as a symmetric dimer with both subunits displaying identical conformational states (Fig. 2A and B). However, it appears that many of the residues from the C-terminus of the regulator are not visible in the structure, probably due to the flexible nature and small size of the regulator. We finally refined cryo-EM structures of this apo-CosR dimer to a resolution of 3.77 Å (Fig. 2B; Table S1). The final model of each subunit contains residues 1–120, 126–143, and 151–161.

The cryo-EM structure of apo-CosR indicates that CosR can be divided into two domains, an N-terminal receiver domain (RD) and a C-terminal DNA-binding domain (DBD), similar to those found in the X-ray structures of the *Thermotoga maritima* DrrB (34), *T. maritima* DrrD (35), and *Mycobacterium tuberculosis* MtrA (36) regulators. Residues 1–116 create the RD, whereas residues 124–221 form the DBD. All of the N-terminal RD residues are clearly visible in the apo-CosR structure; however, many of the C-terminal DBD residues, such as residues 162–221, are imperceptible with no observable cryo-EM densities. Because of the substantial number of missing residues, the numerical assignments of the secondary structures of apo-CosR are based on the secondary structural assignments of the X-ray structures of the DNA-bound CosR regulator (described below). Therefore, the α helices and β strands are designated as follows: α1 (11–23), α2 (32–42), α3 (60–66), β4 (77–79), α4 (84–93), β5 (98–100), α5 (105–113), and α6 (152–160). Residues 117–123 likely form a flexible linker region to directly connect the RD and DBD. This flexible linker can easily change its conformation to accommodate different conformational states of the CosR protomer. Based on the structural information, apo-CosR is very flexible in nature, particularly the C-terminal DBD.

### X-ray structures of the CosR-DNA_1_ complex

Our cryo-EM data strongly indicate that it is possible to obtain detailed structural information of the apo-CosR regulator and CosR-DNA complex, as we can observe cryo-EM maps of both apo and DNA-bound CosR in the cryo-EM grid (Fig. S1). To try and obtain high-resolution structures of this regulator both in the apo form and bound with promotor DNA, we turned to the technique of X-ray crystallography. Vapor diffusion crystallization drops were set up by mixing 10–20 mg/mL purified CosR with well solutions from various commercialized crystallization screening kits. Unfortunately, extensive crystallization trials did not allow us to obtain high-quality crystals suitable for X-ray structural determination.

**Fig 3:**
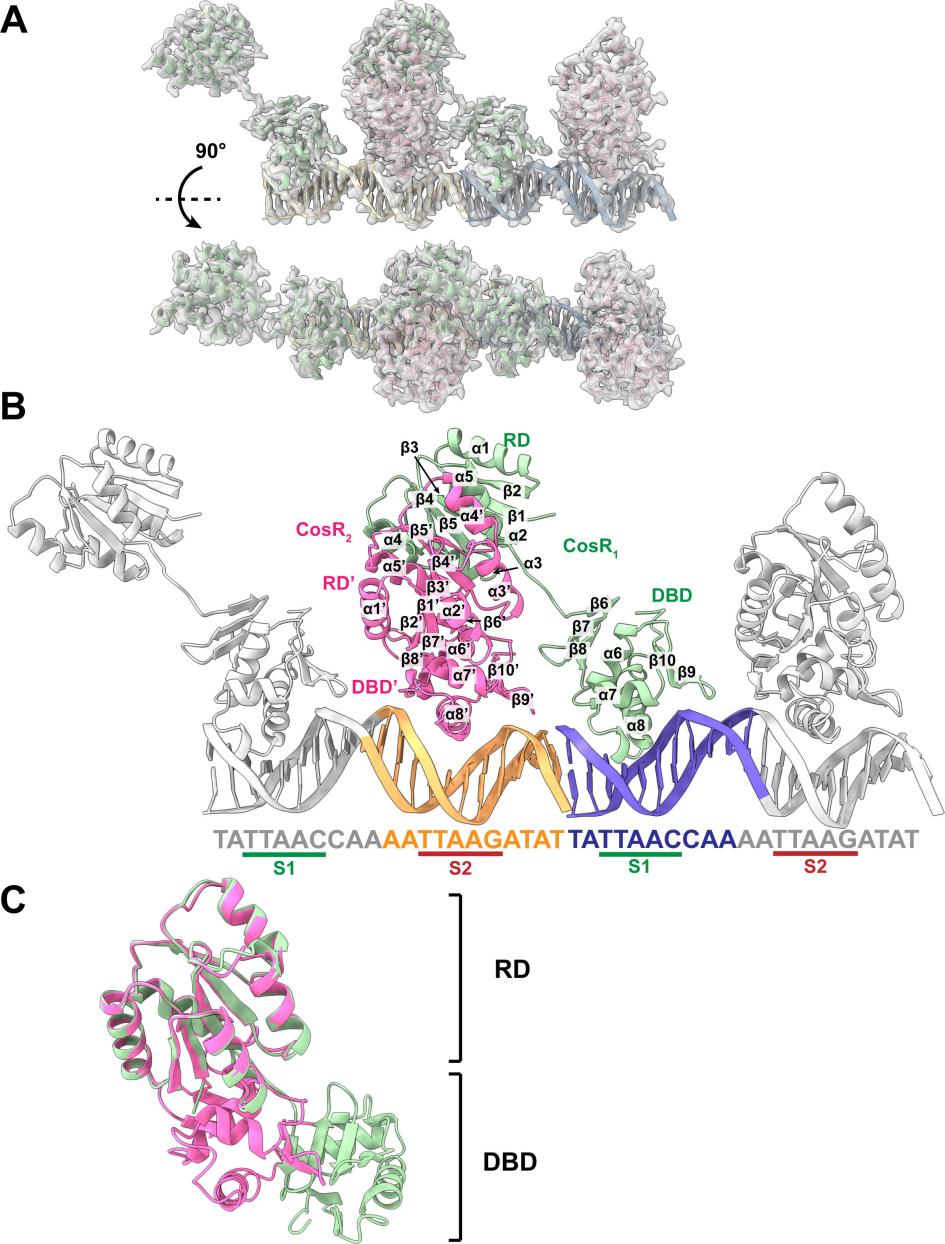
X-ray structure of CosR-DNA_1_. (**A**) Electron density map (2F_o_–F_c_) of the CosR-DNA_1_ complex within two-unit cells of the crystal. The data allow us to determine the crystal structure of CosR-DNA_1_ to a resolution of 2.90 Å. The secondary structural elements of CosR_1_ and CosR_2_ are colored green and pink, respectively. The two DNA_1_ duplexes within two-unit cells are colored slate and orange. (**B**) Ribbon diagram of the crystal structure of the CosR-DNA_1_ complex. The secondary structural elements forming dimeric CosR-DNA_1_ within the two-unit cells are colored (green, CosR_1_; pink, CosR_2_; gray (left), a portion of the DNA_1_ duplex containing the S1 site; orange, a portion of the DNA_1_ duplex containing the S2 site; blue, a portion of the DNA1 duplex containing the S1 site; gray (right), a portion of the DNA1 duplex containing the S2 site), whereas the remaining two other CosR molecules are colored gray. (**C**) Superimposition of the structures of CosR_1_ and CosR_2_. The secondary structural elements of CosR_1_ and CosR_2_ are colored green and pink, respectively. This superimposition indicates that the two CosR molecules are very distinct with a root-mean-square deviation of 9.5 Å.

Next, 8 mg/mL of purified full-length CosR was mixed with 2.6 mg/mL of 21 bp double-stranded DNA_1_, which contains two half-sites, S1 and S2, for dimeric CosR binding, and crystallization trials were again conducted. These two half sites are separated by five bases so that one end of the 21 bp DNA_1_ sequence covers S1 and an extension of two bases. The other end of this DNA_1_ sequence contains S2 and an extension of four bases at this end. The best crystal of the CosR-DNA_1_ complex diffracted X-rays to 2.90 Å. The final X-ray structure of the CosR-DNA_1_ complex was determined at this resolution (Fig. 3A and B; Table S1).

Each unit cell of the CosR-DNA_1_ crystal contains two CosR protomers and one DNA duplex. One of the CosR protomers is bound at S1 of one DNA chain. The other CosR protomer in the unit cell is associated with S2 of the DNA chain (Fig. 3B). It appears that the structures of these two CosR protomers are very different from each other. Superimposition of these two CosR protomers gives rise to a very high root-mean-square deviation (r.m.s.d.) of 9.5 Å, suggesting that they are structurally very distinct from each other (Fig. 3C). The CosR protomer, designated CosR_1_, bound by the S1 site, has an elongated and extended conformation. However, the other CosR protomer that contacts the S2 site, designated CosR_2_, has a more compact conformation. These two CosR molecules are observed to independently bind to the DNA duplex, and they do not seem to interact with each other in the unit cell. However, by applying a crystallographic symmetry operator, it is found that CosR_1_ and CosR_2_ indeed tightly contact with each other and assemble as an asymmetric dimer to bind the DNA duplex (Fig. 3B). This observation suggests that CosR should be in the form of a dimer to specifically interact with the target DNA. Interestingly, this CosR dimer appears to hold the two DNA_1_ chains together, where the two DNA_1_ align in such a way that the tail of the first chain contacts the head of the second chain. This arrangement seems to allow these two DNA_1_ chains to assemble as a single 42 bp DNA duplex within two-unit cells (Fig. 3B).

Similar to the structure of apo-CosR, each CosR protomer of the CosR-DNA_1_ complex consists of an N-terminal RD (residues 1–116) and a C-terminal DBD (residues 124–221). These two domains are directly connected by a flexible linker (residues 117–123). Within each protomer, the RD is made up of five α helices and five β sheets, whereas the DBD is composed of three α helices and five β sheets. Based on the structural information, the RD is responsible for forming a dimer interface, securing the dimeric oligomerization. However, the two DBDs within the dimer create interaction sites for anchoring the DNA duplex to control gene regulation.

The α helices and β strands are designated numerically from the N- to C-termini: β1 (2–7), α1 (10–23), β2 (26–30), α2 (33–42), β3 (47–51), α3 (59–69), β4 (74–79), α4 (84–93), β5 (97–101), α5 (105–116), β6 (124–126), β7 (129–132), β8 (136–140), α6 (150–160), α7 (168–175), α8 (184–199), β9 (207–210), and β10 (213–217).

### X-ray structures of the CosR-DNA_2_ complex

**Fig 4:**
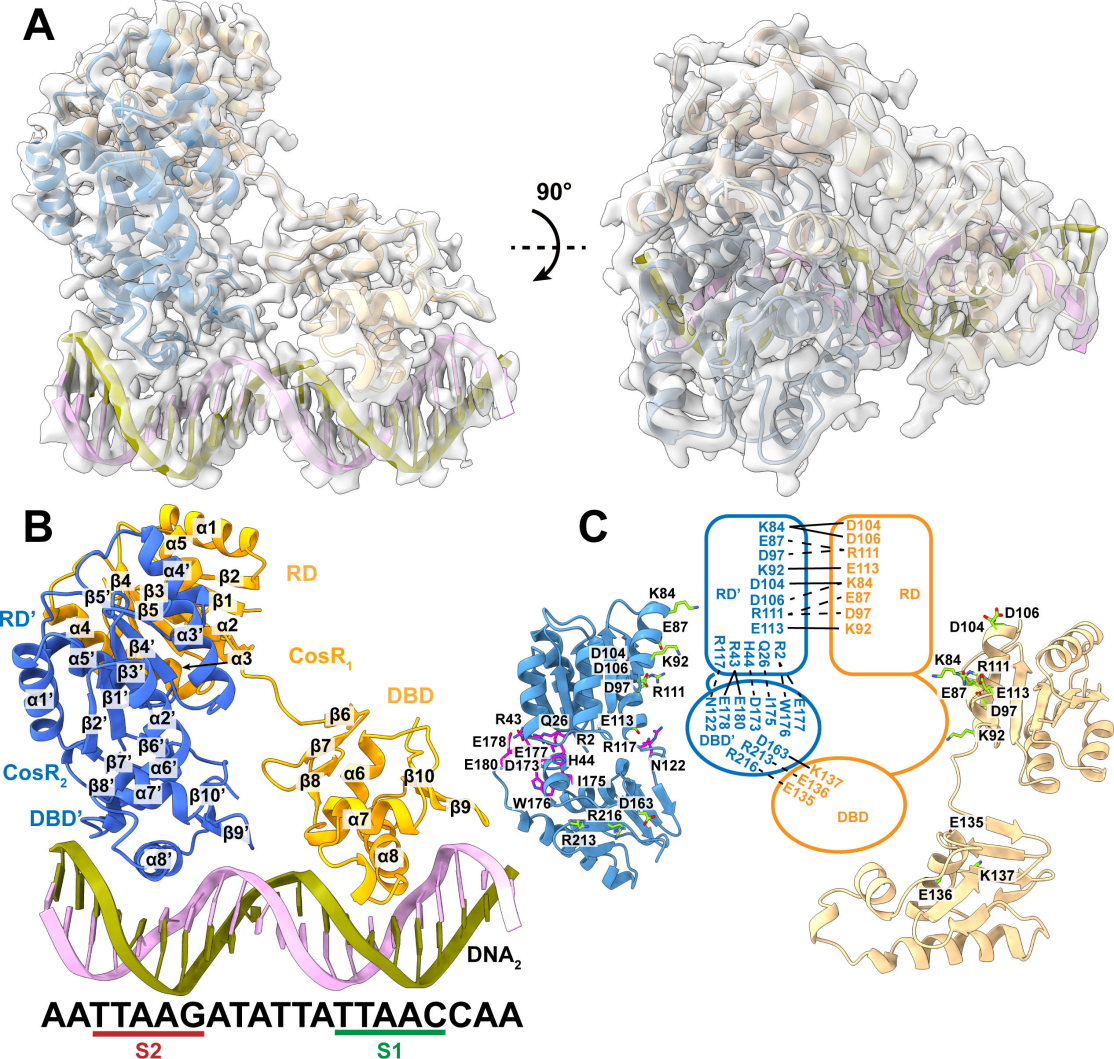
X-ray structure of CosR-DNA_2_. (**A**) Electron density map (2F_o_–F_c_) of the CosR-DNA_2_ complex within a unit cell of the crystal. The data allow us to determine the crystal structure of CosR-DNA_2_ to a resolution of 2.20 Å. The secondary structural elements of CosR_1_ and CosR_2_ are colored orange and blue, respectively. The DNA_2_ duplex is colored dark green and light pink. (**B**) Ribbon diagram of the crystal structure of the CosR-DNA_1_ complex. The secondary structural elements of CosR_1_ and CosR_2_ are colored orange and blue. The DNA_2_ duplex is colored dark green and light pink. (**C**) Intra- and intersubunit interactions of dimeric CosR. CosR_1_ is colored orange, and CosR_2_ is colored blue in this cartoon. The intra- and intersubunit hydrogen bonds and salt bridges are represented by black dotted and black solid lines. Ribbon diagrams of the CosR_1_ (blue) and CosR_2_ (orange) subunits are also included in this figure. Important CosR residues for inter- and intrasubunit interactions are highlighted with light green and magenta sticks.

Based on the structural information of the CosR-DNA_1_ complex, we rationalized that we could make the CosR dimer bind one single DNA duplex by flipping the DNA sequence AATTAAGATAT, which belongs to the second half of the DNA_1_ chain, to the front of the first half (TATTAACCAA) of the sequence to form the new 21 bp DNA, AATTAAGATATTATTAACCAA (DNA_2_). Fluorescence polarization indicates that the new DNA_2_ duplex also specifically binds CosR within the nanomolar range (Fig. 1B). The CosR-DNA_2_ complex was then crystallized using the same vapor diffusion approach. The best crystal of the CosR-DNA_2_ complex diffracted X-rays to 2.20 Å, which allowed us to determine the X-ray structure of the CosR-DNA_2_ complex at this resolution (Fig. 4A and B; Table S1).

The crystal structure of the CosR-DNA_2_ indeed depicts that the CosR dimer contacts one DNA_2_ duplex to form this regulator-promoter complex (Fig. 4B). Also, the extended protomer CosR_1_ was observed to bind to the S1 site, whereas the compact protomer CosR_2_ interacts with the S2 site to form this complex (Fig. 4B). Overall, the structure of CosR-DNA_2_ is very similar to that of CosR-DNA_1_. Superimposition of the two CosR dimers from these two structures gives rise to an r.m.s.d. of 0.38 Å, suggesting that the conformational states of these two dimers are nearly identical to each other.

The dimer interface of CosR is predominately created by α4, α5, β4, and loops connecting these structural elements of each N-terminal RD of the CosR protomer. Strong interactions at this interface secure the dimeric oligomerization. There are at least five hydrogen bonds and five salt bridges involved in dimerization at this interface (Fig. 4C). In addition, helices α4 and α5′ (as well as helices α5′ and α4) are approximately 6 Å apart, performing coiled-coil interactions to further secure the dimeric assembly.

Within the asymmetric CosR dimer, the CosR_1_ protomer features an extended conformation, whereas the CosR_2_ protomer displays a compact form of this protein. Extensive interactions between the RD and DBD of CosR_2_ create this compact conformation. It is observed that at least five hydrogen bonds and two salt bridges participate in RD and DBD interactions to secure this compact CosR_2_ protomer (Fig. 4C). These interactions are absent in the extended form of the CosR_1_ protomer. In addition, the two DBDs within the CosR dimer made two hydrogen bonds and one salt bridge at the interface between these two DBDs to help stabilize this asymmetric dimeric structure (Fig. 4C).

**Fig 5:**
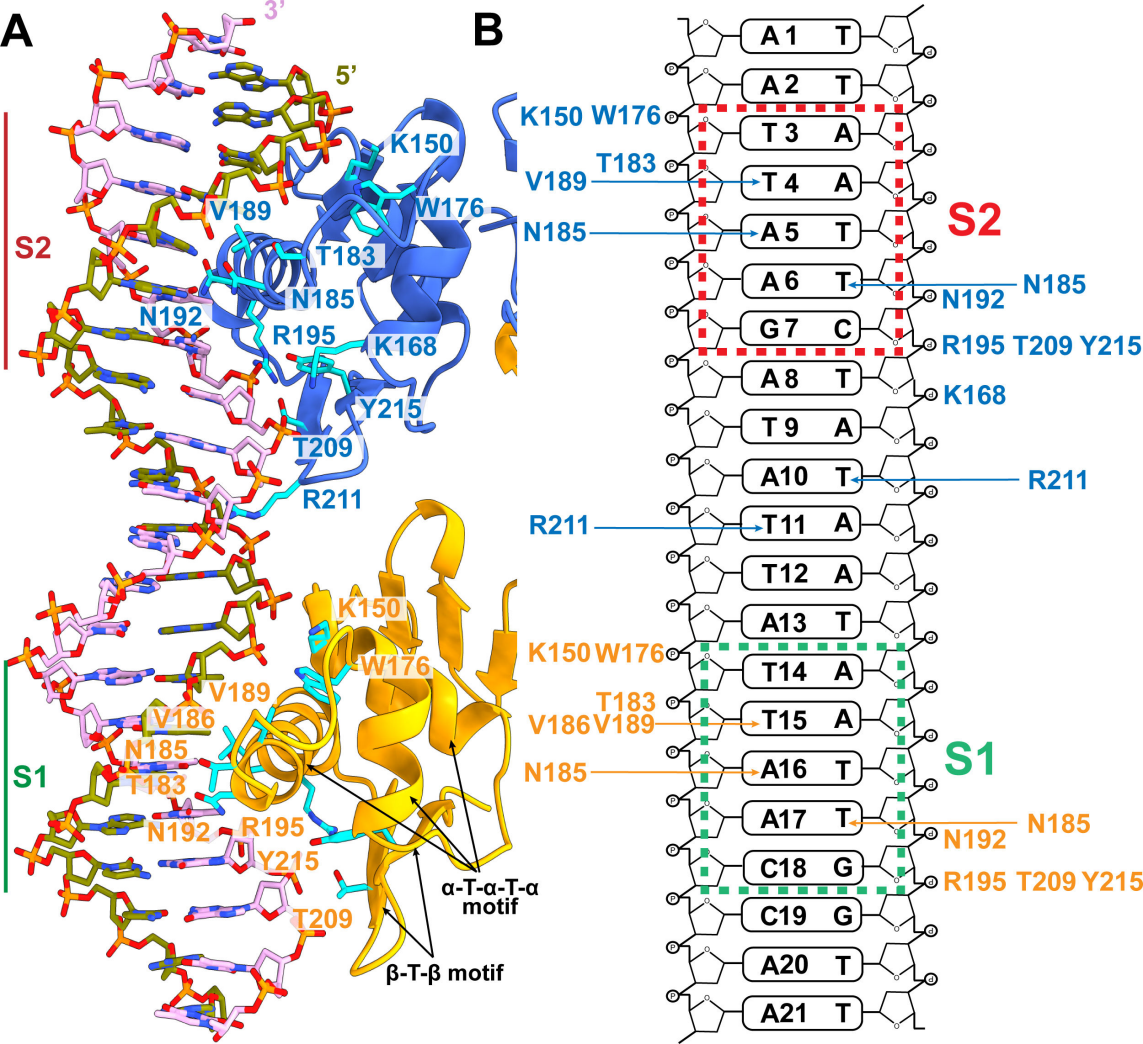
CosR and DNA_2_ interactions. (**A**) Ribbon diagram of the DBDs of CosR_1_ and CosR_2_ and the 21 bp DNA_2_ duplex. The diagram depicts that helix-turn-helix-turn-helix motifs of CosR_1_ (orange) and CosR_2_ (blue) directly contact the major grooves at the S1 and S2 sites of DNA_2_, whereas the beta-turn-beta motifs of CosR_1_ (orange) and CosR_2_ (blue) specifically interact with the minor grooves of DNA_2_. Within 3.5 Å, the CosR residues that directly interact with nucleotides of the 21 bp DNA_2_ are highlighted as cyan sticks. (**B**) Contact map for CosR and DNA_2_ interactions. Residues within 3.5 Å of the DNA_2_ nucleotides are listed. The S1 and S2 sites are boxed.

Interestingly, the C-terminal DBD constitutes a typical helix-turn-helix-turn-helix motif, containing helices α6–α8 to contact the major groove of the DNA duplex. In addition, a beta-turn-beta wing motif made up of β9 and β10 interacts with the minor groove of the promotor DNA to further secure regulator-promotor binding. Specific interactions between target DNA and the CosR dimer are shown in Fig. 5.

### Conformational flexibility of the CosR regulator

**Fig 6:**
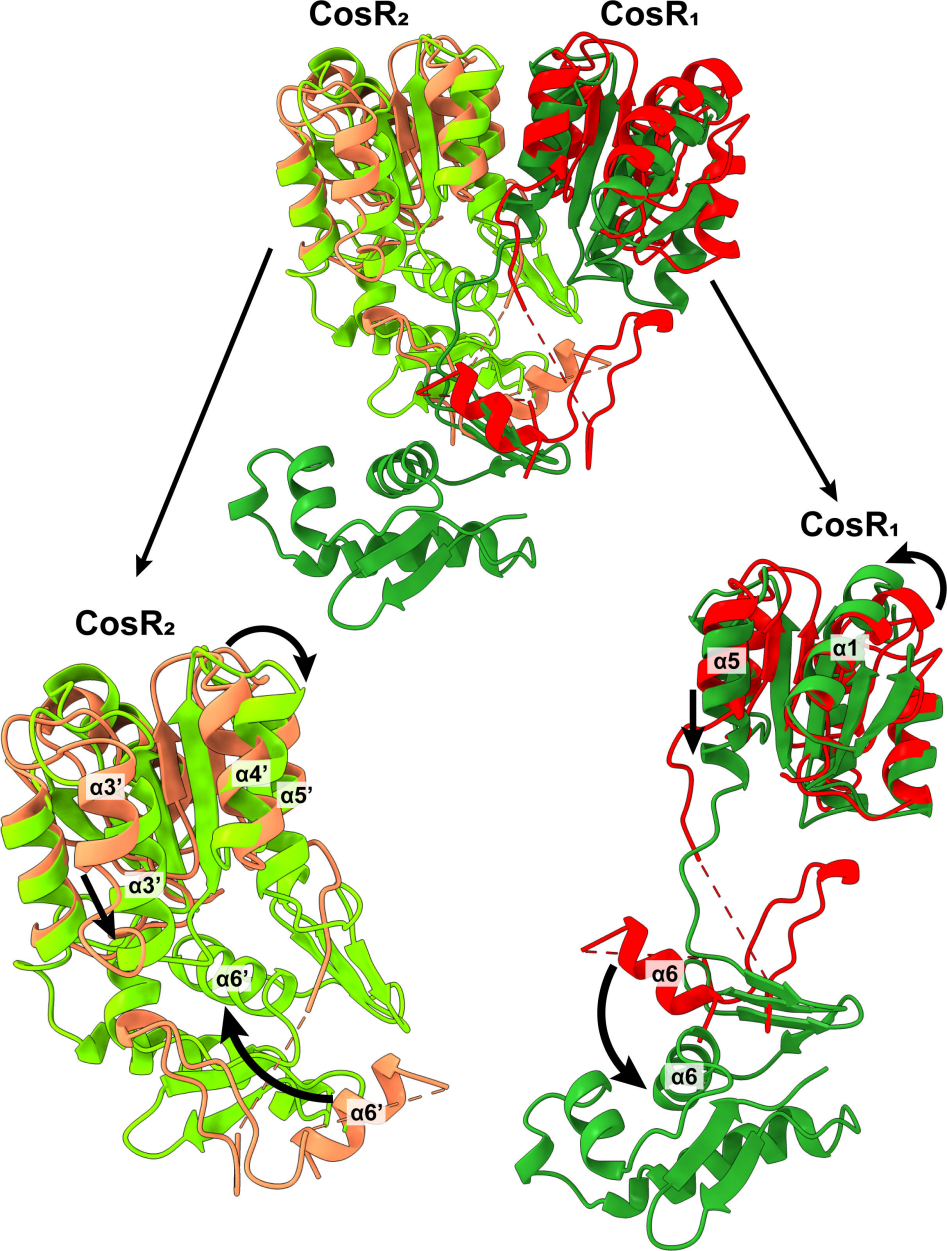
Superimposition of the structure of the CosR dimer from CosR-DNA_2_ to that of the apo-CosR dimer. This superimposition indicates that the structures of the CosR dimer from CosR-DNA_2_ are very distinct from that of apo-CosR with an r.m.s.d. of 9.5 Å. The CosR_1_ and CosR_2_ molecules of the CosR-DNA_2_ complex are colored dark green and light green. Their corresponding CosR molecules within the apo-CosR dimer are colored red and orange. Individual superimpositions of CosR_1_ with its corresponding apo-CosR monomer and CosR_2_ with its corresponding apo-CosR monomer give rise to r.m.s.d. values of 6.9 and 11.5 Å. These pairwise superimpositions depict that there are substantial changes in relative positions of α1, α5, α3′, α4′, and α5′ of the RD, and α6 and α6′ of the DBD when compared with the secondary structural elements of CosR-DNA_2_ and apo-CosR.

When the structures of the symmetric dimer of apo-CosR and the asymmetric dimer of CosR-DNA_2_ were compared, it was found that each apo-CosR molecule was very distinct from those of the compact state of CosR_2_ and extended state CosR_1_ in the DNA-bound form. Superimpositions of a molecule of apo-CosR to CosR_1_ and CosR_2_ provide large r.m.s.d. values of 6.9 and 11.5 Å, indicating these three CosR molecules represent very different conformational states of the regulator (Fig. 6). These superimpositions also allow us to postulate as to how each CosR molecule switches in conformation within the dimer to accommodate for the binding of the target DNA.

The superimposition of a monomer of apo-CosR to CosR_2_ depicts that there are substantial conformational differences within the N-terminal RD, linker region, and C-terminal DBD of these two conformers. For example, helix α5′, which directly connects the flexible linker between the RD and DBD, seems to shift in position in the CosR_2_ protomer when compared with the structure of the apo-CosR protomer. The C-terminal of helix α5′ performs a rigid-body tilt by 25° to shift the conformation from the apo to DNA-bound form. It is observed that helices α3′ and α4′ also make similar changes to accommodate for the movement of helix α5′. These changes in conformation may allow the linker to flip the location of the entire C-terminal DBD from the left to the right side (the orientation is based on Fig. 6). At this point, helix α6′, which forms part of the helix-turn-helix-turn-helix motif for DNA recognition, has drastically altered its position and orientation to accommodate for DNA binding (Fig. 6). It can be interpreted that helix α6′ performs a rigid-body translational movement to shift its position to the right by 16 Å. The C-terminal end of helix α6 also participates in a 145° rigid-body rotation to shift its orientation from the apo to DNA-bound structures.

Superimposition of the other monomer of apo-CosR to CosR_1_ also illustrates that helix α5 makes a substantial movement to shift its conformation from the apo to DNA-bound forms. This change can be interpreted as a combination of a 5 Å rigid body shift toward the target DNA and a 15° rigid-body rotation of the C-terminus of this helix. In addition, helix α1 also adjusts its position to accommodate the change of helix α5. The net result is that helix α6, which contributes to form the helix-turn-helix-turn-helix motif, appears to shift toward the target DNA by 7 Å. It also allows this helix to adjust its orientation by performing a 90° rigid-body rotation of the entire helix to accommodate for DNA binding (Fig. 6). All of these conformational changes are substantial, particularly within the C-terminal DBD, to facilitate target DNA recognition and regulate protein expression.

CosR in *C. jejuni* is a nontypical response regulator as it lacks a cognate sensor kinase (20). How the signal is transduced to CosR remains unknown. Although a sensor kinase encoding gene (*cosS*) exists immediately upstream of *cosR in Campylobacter fetus*, *cosS* is absent in *C. jejuni* and other thermophilic *Campylobacter* species (20). Additionally, there is a D51N mutation in the CosR of *C. jejuni* compared with that of *C. fetus*. Furthermore, the CosS kinase of *C. fetus* is a functionally active autokinase but failed to phosphorylate CosR of *C. jejuni* even after N51 was mutated back to D51 (26). These observations suggest that the RD of *CosR* in *C. jejuni* lost the ability to conduct the phosphoryl relay. How CosR receives signals and how these signals affect its binding to target DNA remains unknown. The structural information generated in this study has provided hints for the molecular mechanisms of this regulator. The data strongly suggest that CosR is a highly flexible protein. The flexibility and plasticity of this regulator may promote a long-distance conformational coupling between the N-terminal RD and C-terminal DBD to receive signals from the outside of the cell, in turn, facilitating the binding of DBD to the promotor DNA for regulating gene expression. It appears that regulators of the OmpR/PhoB family are intrinsically quite flexible, which can be clearly depicted by comparing the DNA-bound structures of the *Escherichia coli* KdpE (37) and *Klebsiella pneumoniae* PmrA (38) dimeric regulators with that of *C. jejuni* CosR (Fig. S2). This superimposition indeed highlights the conformational flexibility and diversity of this class of regulators and their ability to accommodate these regulators to bind their target DNAs.

Several crystal structures of the DNA unbound state of the OmpR/PhoB family of regulators have been reported, including *T. maritima* DrrB (34), *T. maritima* DrrD (35), and *M. tuberculosis* MtrA (36). The oligomerization state of these DNA-free regulator structures is largely monomeric, leading to a proposed mechanism where the first step toward the formation of the regulator-DNA complex is dimerization of the regulator. However, our cryo-EM structure of apo-CosR indicates that CosR is in a dimeric form in solution. These cryo-EM images lead us to postulate that CosR may prefer this dimeric oligomerization state in its native cellular environment. Based on the structures of both apo-CosR and CosR-DNA, it appears that the interactions between the two CosR protomers at the dimer interface are substantial. This strengthens our hypothesis that CosR prefers its dimeric form in aqueous solution. Indeed, our X-ray structure of the CosR-DNA_1_ complex indicates that the strength of protomer–protomer interaction at the dimer interface is strong enough to allow the CosR dimer to hold two DNA chains at a time.

### Summary

**Fig 7:**
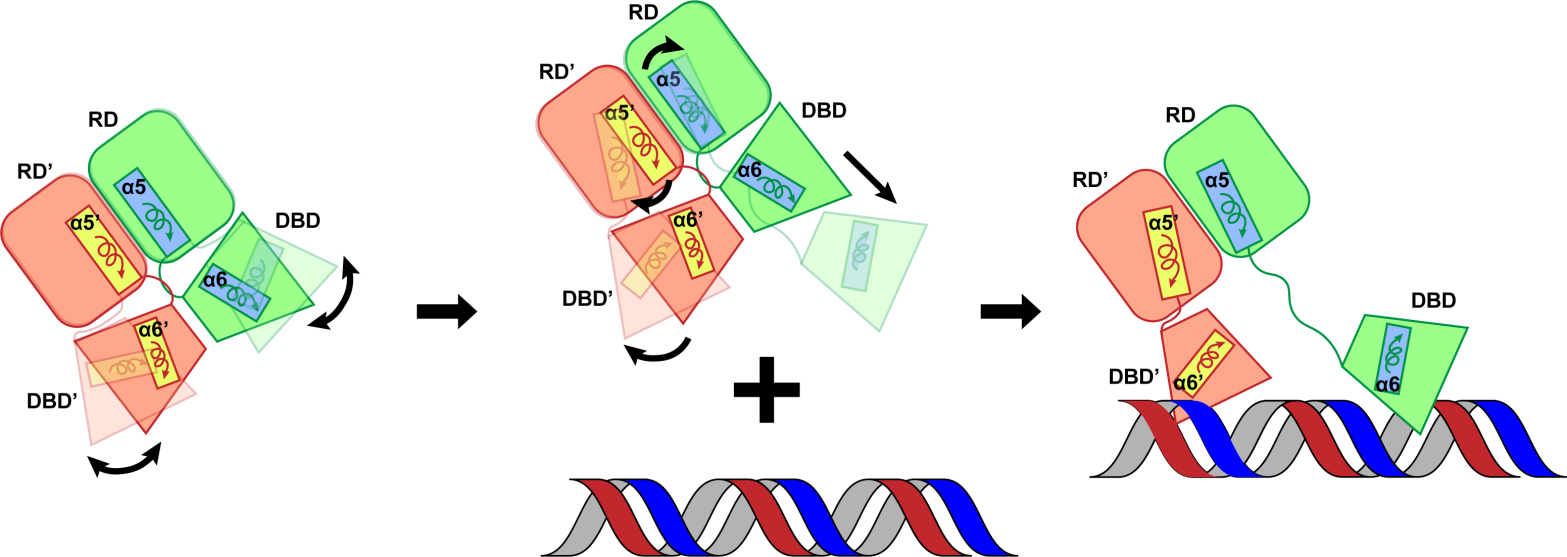
Proposed mechanism for DNA binding by CosR. The DNA-free form of CosR resides in its dimeric oligomerization state within the cell. However, the C-terminal DBD is very flexible. The binding of the target DNA involves a series of rigid-body translational and rigid-body rotational movements. The secondary structural elements of the N-terminal RD of each CosR protomer slightly adjust their position and orientation to facilitate DNA recognition, but the C-terminal DBD undergoes a substantial rearrangement of its secondary structural elements in order to accommodate DNA binding.

Based on the structural information from both cryo-EM and X-ray crystallography, we propose an induced fit mechanism that leads to the recognition and binding of the CosR dimer to promotor DNA (Fig. 7). First, the DNA-free form of CosR resides in its dimeric oligomerization state within the cell. The binding of the target DNA involves a series of rigid-body translational and rigid-body rotational movements of different domains of the dimeric CosR regulator, allowing the dimer to fit into the major and minor grooves of the target DNA. The secondary structural elements of the N-terminal RD of each CosR protomer slightly adjust their position and orientation to facilitate DNA recognition. In contrast, the C-terminal DBD undergoes a very large transition that involves the rearrangement of its secondary structural elements in order to accommodate DNA binding. It was observed from our crystal structure analysis of CosR-DNA that helix α8 of the helix-turn-helix-turn-helix motif from each CosR protomer is completely buried in the major groove of the DNA duplex and makes substantial contacts to bind DNA. Additionally, β9 and β10, along with the flexible loop between them that forms a beta-turn-beta wing motif directly, interact with the minor groove of target DNA to fine-tune and strengthen regulator–promotor interactions.

## MATERIALS AND METHODS

### Expression and purification of CosR

Full-length *C. jejuni* CosR protein with a 6×His tag at the C-terminus was cloned into pET15b to create the expression vector pET15bΩ*cosR*. The plasmid was transfected into *E. coli* BL21(DE3) cells to overproduce the CosR protein. Cells were grown in 1 L of Luria-Bertani medium supplemented with 100 µg/mL ampicillin at 37°C. When the OD_600_ reached 0.5, the expression of CosR was induced with 0.2 mM isopropyl-β-D-thiogalactopyranoside. Cells were then harvested within 4 hours of induction. The collected bacterial cells were resuspended in a buffer containing 20 mM Na-HEPES (pH 7.5), 500 mM NaCl, and 1 mM phenylmethanesulfonyl fluoride and disrupted using a French pressure cell. Cell debris was removed by centrifugation at 186,000 × g and 4°C for 45 min. The soluble fraction was collected, and DNase I was added to a final concentration of 1 µM. The CosR protein and DNase I mixture were incubated on ice for 30 min and then purified with a Ni^2+^-affinity column. Purified CosR was dialyzed against a buffer containing 20 mM Na-HEPES (pH 7.5) and 100 mM NaCl and then concentrated to 20 mg/mL. Subsequently, the protein was further purified using a Superdex 200 column (GE Healthcare) equilibrated with 20 mM Na-HEPES (pH 7.5) and 100 mM NaCl. Fractions corresponding to the purified CosR protein were collected and concentrated to 20 mg/mL (785 µM).

### Fluorescence polarization assay for the DNA binding affinities

Fluorescence polarization assays were used to determine the DNA binding affinities of the CosR regulator. Both the 21 bp oligodeoxynucleotide and fluorescein-labeled oligodeoxynucleotide were purchased from Thermo Fisher Scientific, Inc. (Waltham, MA). These oligodeoxynucleotides contain the S1 and S2 sites for CosR binding. The DNA_1_ sequences are 5′-ATATCTTAATTTTGGTTAATA-3′ and 5′-F-TATTAACCAAAATTAAGATAT-3′, where F denotes the fluorescein that was covalently attached to the 5′ end of the oligodeoxynucleotide by a hexamethylene linker. The DNA_2_ sequences are 5′-TTGGTTAATAATATCTTAATT-3′ and 5′-F-AATTAAGATATTATTAACCAA-3′. Each 21 bp fluoresceinated ds-DNA was prepared by annealing the two corresponding oligodeoxynucleotides together at 95°C for 5 min. Fluorescence polarization experiments were done using a DNA binding solution containing 10 mM phosphate-buffered saline (PBS) (pH 7.4), 5 nM fluoresceinated DNA_1_ or DNA_2_, and 1 µg of poly(dI-dC) as nonspecific DNA_1_ or DNA_2_. The protein solution containing 150 nM dimeric CosR and 5 nM fluoresceinated DNA_1_ or DNA_2_ was titrated into the DNA binding solution until the millipolarization became unchanged. All measurements were performed at 25°C using a PerkinElmer LS55 spectrofluorometer equipped with a Hamamatsu R928 photomultiplier. The excitation wavelength was 490 nm, and the fluorescence polarization signal (in ΔP) was measured at 520 nm. Each titration point recorded was an average of 15 measurements. The titration experiments were repeated three times to obtain the average K_D_ value. Curve fitting was accomplished using the program ORIGIN (OriginLab Corporation, Northampton, MA, USA).

### Cryo-EM sample preparation

For imaging CosR-DNA, a sample containing 20 µM CosR and 10 µM 40 bp ds-DNA duplex (containing 5′-AATAATTTTATTAACCAAAATTAAGATATAATTAGCCAAA-3′ and 5′-TTTGGCTAATTATATCTTAATTTTGGTTAATAAAATTATT-3′) was directly applied to glow-discharged holey carbon grids (Quantifoil Cu R1.2/1.3, 300 mesh), blotted for 18 s, and then plunge-frozen in liquid ethane using a Vitrobot (Thermo Fisher). All grids were then transferred into cartridges prior to data collection.

### Cryo-EM data collection

The cryo-EM images were collected in super-resolution mode at 165 K magnification on a Titan Krios equipped with a K3 direct electron detector (Gatan, Pleasanton, CA). The physical pixel size was 0.666 Å/pix (super-resolution of 0.333 Å/pix). Each micrograph was exposed to a total dose of 54.6 e^-^/Å^2^ for 4 s, and 30 frames were captured using SerialEM (39).

### Cryo-EM data processing

The super-resolution image stack was aligned and binned by two using patch motion. The contrast transfer function (CTF) was estimated using patch CTF in cryoSPARC (40). Blob Picker followed by 2D classification was used to generate templates for automated template picking. Initially, 1,504,675 particles were selected after auto picking in cryoSPARC. Several iterative rounds of 2D classifications followed by *ab initio* and heterogeneous 3D classifications were performed to remove false picks and classes with unclear features, ice contamination, or carbon. The 3D classification analysis was then employed, resulting in three distinct classes of images. A single round of nonuniform refinement followed by local refinement with nonuniform sampling resulted in 3.77, 7.71, and 6.61 Å resolution cryo-EM maps for apo-CosR, CosR-DNA complex, and free DNA based on the gold standard Fourier shell correlation (FSC 0.143; Fig. S1).

### Model building and refinement

Model building of the apo-CosR dimer was based on the cryo-EM map. The model of the N-terminal RD was predicted using AlphaFold (41). This predicted model was used as a starting model for molecular replacement to determine the CosR structure. The subsequent model rebuilding was performed using COOT (42). Structural refinements were performed using the phenix.real_space_refine program from the PHENIX suite (43). The final atomic model was evaluated using MolProbity (44). The statistics associated with data collection, 3D reconstruction, and model refinement are included in Table S1.

### Crystallization and X-ray data collection

For crystallizing the CosR-DNA_1_ complex, a mixture consisting of 1 µL of CosR-DNA_1_ solution (8 mg/mL CosR and 2.6 mg/mL 21 bp DNA_1_ duplex) and 1 µL of well solution (0.02 M sodium formate, 0.02 M ammonium acetate, 0.02 M sodium citrate, 0.02 M sodium potassium tartrate, 0.02 M sodium oxamate, 0.01 M Na-MES [pH 6.5], 40% [vol/vol] PEG 500 MME, and 20% [wt/vol] PEG 20,000) was equilibrated against 80 µL of well solution using vapor diffusion. Crystals of the CosR-DNA_1_ complex appeared in the drop within 2 weeks. Similarly, for CosR-DNA_2_ crystallization, a 2 µL drop consisting of 1 µL of CosR-DNA_2_ solution (320 µM CosR and 192 µM 21 bp DNA_2_ duplex) and 1 µL of well solution (0.03 M magnesium chloride, 0.03 M calcium chloride, 0.1 M Na-MES [pH 6.5], 40% [vol/vol] ethylene glycol, and 20% [wt/vol] PEG 8,000) was equilibrated against 80 µL of well solution using vapor diffusion. Crystals of CosR-DNA_2_ appeared in the drop within 2 weeks. The crystallization conditions of both CosR-DNA_1_ and CosR-DNA_2_ provided sufficient cryoprotection. These crystals were directly harvested and frozen in liquid nitrogen at 100 K. Diffraction data sets were taken from the Advanced Light Source (beamline 24-ID-C) at cryogenic temperature (100K) using a DECTRIS EIGER2 × 16M detector. Data were processed using HKL2000 (45). The crystallographic data and statistics are given in Table S1.

### Crystal structural determination and refinement

Molecular replacement was used to determine the structure of CosR-DNA_1_, utilizing a molecule of apo-CosR as the template. This approach allowed us to successfully trace two molecules of the N-terminal RD of CosR in the asymmetric unit. Iterative model building and density modiﬁcation were carried out using PHENIX (43) and COOT (42). The double-stranded DNA was built manually based on the 2F_o_–F_c_ electron density map using COOT (42). Structure reﬁnement was performed using PHENIX (43). Similarly, structural determination of the CosR-DNA_2_ complex was performed by molecular replacement, utilizing the CosR-DNA_1_ structure as the template. Model refinement was carried out using PHENIX (43) and COOT (42). The reﬁnement statistics are reported in Table S1.

## Data Availability

X-ray structures and atomic coordinates of CosR-DNA_1_ and CosR-DNA_2_ have been deposited with PDB accession codes 8UVX and 8UVK. Cryo-EM structural information and density map of CosR have been deposited with accession codes 8UUZ (PDB) and EMD-42602 (EMDB).

## SUPPLEMENTAL MATERIAL

Fig. S1 mbio.03430-23-s0001.jpeg CosR cryo-EM data processing.

Fig. S2 mbio.03430-23-s0002.jpeg Data collection and structural refinement statistics.

Supplemental Text mbio.03430-23-s0003.docx Captions of Fig. S1, Fig. S2, and Table S1.

Table S1 mbio.03430-23-s0004.docx Data collection and structural refinement statistics.

ASM does not own the copyrights to Supplemental Material that may be linked to, or accessed through, an article. The authors have granted ASM a non-exclusive, world-wide license to publish the Supplemental Material files. Please contact the corresponding author directly for reuse.
