# Supplementary figures and images for: Structural basis of DNA recognition of the Campylobacter jejuni CosR regulator
Source: mBio. 2024 Feb 7;15(3):e03430-23. doi: 10.1128/mbio.03430-23 (PMC10936212; doi:10.1128/mbio.03430-23)

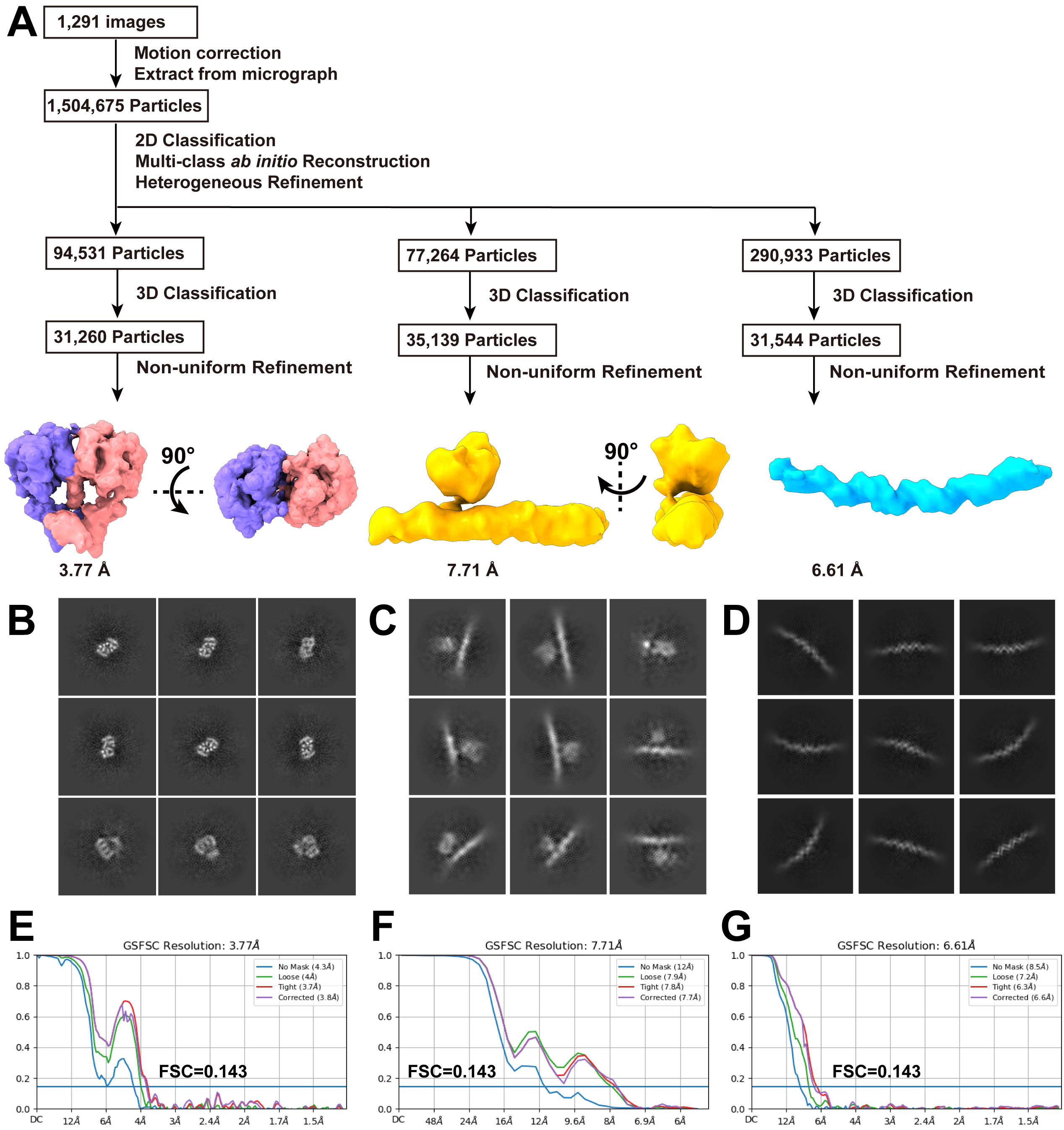

Supplement: Fig. S1 — CosR cryo-EM data processing. [file mbio.03430-23-s0001.jpeg]

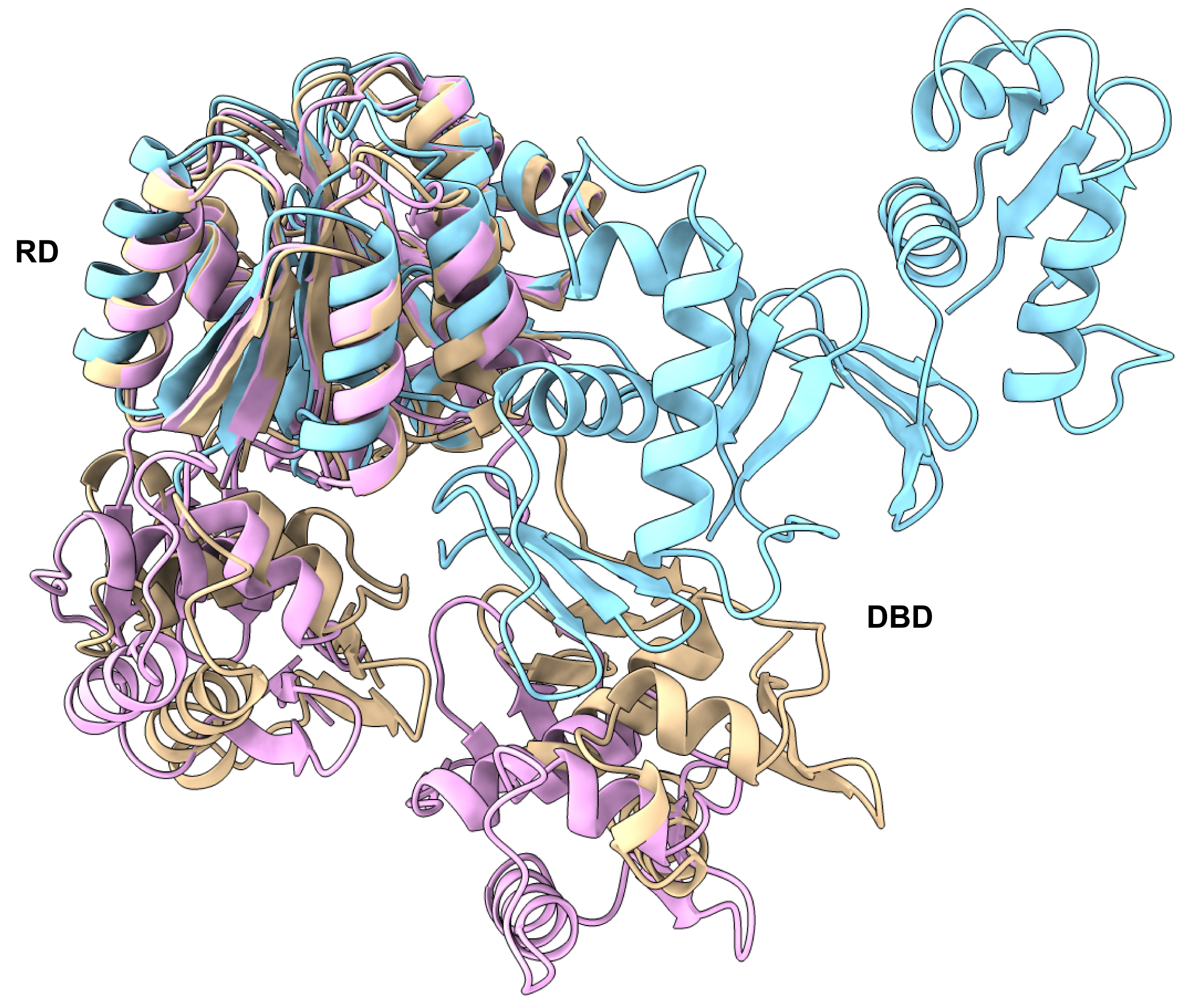

Supplement: Fig. S2 — Data collection and structural refinement statistics. [file mbio.03430-23-s0002.jpeg]
