# Supplementary material for: Structural basis of DNA recognition of the Campylobacter jejuni CosR regulator
Source: mBio. 2024 Feb 7;15(3):e03430-23. doi: 10.1128/mbio.03430-23 (PMC10936212; doi:10.1128/mbio.03430-23)
Supplement: Supplemental Text — Captions of Fig. S1, Fig. S2, and Table S1. [file mbio.03430-23-s0003.docx]

**SUPPLEMENTAL FIGURES AND TABLES**

**Fig. S1. CosR cryo-EM data processing.** (A) Data processing workflow of apo-CosR, CosR-DNA and free DNA. Side and top views of the apo-CosR, CosR-DNA and free DNA. (B-D) Representative 2D classes of apo-CosR, CosR-DNA and free DNA. (E-G) Gold-Standard Fourier shell correlation (GS-FSC) curves of apo-CosR, CosR-DNA and free DNA.

**Fig. S2. Structural comparison of the OmpR/PhoB family response regulators**. This is a superimposition of crystal structures of dimeric CosR, KdpE and PmrA in their DNA bound forms. However, their corresponding DNAs were removed from the structures. The CosR, KdpE and PmrA regulators are colored brown, cyan and pink, respectively.

**Table S1.** **Data collection and structural refinement statistics.**
