# Supplementary material for: Structural basis of DNA recognition of the Campylobacter jejuni CosR regulator
Source: mBio. 2024 Feb 7;15(3):e03430-23. doi: 10.1128/mbio.03430-23 (PMC10936212; doi:10.1128/mbio.03430-23)
Supplement: Table S1 — Data collection and structural refinement statistics. [file mbio.03430-23-s0004.docx]

| **Table S1** **Data collection and structural refinement statistics** | | | | |
| --- | --- | --- | --- | --- |
| **Data collection** | **Cryo-EM** | **X-ray** | | |
|  | **apo-CosR** |  | **CosR-DNA_1_** | **CosR-DNA_2_** |
| Magnification | 165,000 | Wavelength (Å) | 0.9792 | 0.9792 |
| Voltage (kV) | 300 | Space group | P 1 21 1 | P 1 21 1 |
| Electron Microscope | Krios-GIF-K3 | Cell constants  a, b, c, α, β, γ | 67.12Å 81.30Å 68.07Å  90.00° 106.14° 90.00° | 66.62 Å 80.10 Å 68.20Å  90° 105.603° 90° |
| Defocus (um) | -0.8 to -1.5 | Resolution (Å) | 45-2.9 | 65.69-2.21 |
| Energy filter width (eV) | 20 | Completeness (%) | 87.4 | 96.38 |
| Pixel size (Å) | 0.666 (0.333) | Total no. of reflections | 13830 | 59474 |
| Total dose (e^-^/ Å^2^) | 54.6 | No. of Unique reflections | 13804 | 33515 |
| Number of frames | 30 | Average I/s | 10.14 | 9.85 |
| Number of micrographs | 1,291 | Data redundancy | 6.9 | 1.8 |
| **Refinement** |  | **Refinement** |  |  |
| Total Particles (no.) | 31,260 | Rwork (%) | 24.1 | 25.3 |
| GS-FSC Resolution (0.143, Å)^a^ | 3.77 | Rfree (%) | 26.8 | 27.9 |
| Model composition |  | Model composition |  |  |
| Chains | 2 | Chains | 2 | 2 |
| Protein residues | 300 | Protein residues | 442 | 440 |
| Ligand | 0 | DNA residues | 42 | 42 |
| r.m.s.d. |  | rms deviations |  |  |
| Bond lengths (Å) | 0.003 | Bond angles (°) | 1.10 | 1.11 |
| Bond angles (°) | 0.804 | Bond length (Å) | 0.008 | 0.008 |
| **Validation** |  | **Validation** |  |  |
| MolProbity score | 2.88 | MolProbity score | 2.11 | 1.86 |
| Clash score | 15.55 | Clash score | 17.21 | 11.94 |
| Ramachandran plot |  | Ramachandran plot |  |  |
| Favored (%) | 95.83 | Favored (%) | 95.21 | 96.08 |
| Allowed (%) | 4.17 | Allowed (%) | 4.57 | 3.92 |
| Disallowed (%) | 0.00 | Disallowed (%) | 0.22 | 0 |
| CC box | 0.59 | CC_1/2_ | 0.980 | 0.996 |
